# Supplementary material for: Drosophila TRPγ is required in neuroendocrine cells for post-ingestive food selection
Source: eLife. 2022 Apr 13;11:e56726. doi: 10.7554/eLife.56726 (PMC9068209; doi:10.7554/eLife.56726)
Supplement: Source data 1. [file elife-56726-data1.docx]

**Figure 1A** Summary statistics.

| Genotypes | n | Means ±SEMs | *P* values |
| --- | --- | --- | --- |
| control | 13 | 0.39 ±0.04 | - |
| *trpγ^1^* | 13 | 0.30 ±0.05 | *P*=0.09 |
| *Dh44* | 6 | 0.33 ±0.06 | *P*=0.39 |
| *Gr64^ab^* | 6 | 0.09 ±0.02 | *P*=4.1 X10^-4^ |

**Figure 1B** Summary statistics.

| Genotypes | n | Means ±SEMs | *P* values  (with control) | *P* values  (with *trpγ^1^*) |
| --- | --- | --- | --- | --- |
| control | 10 | -0.51 ±0.03 | - | - |
| *Gr64^ab^* | 6 | -0.40 ±0.05 | *P*=0.64 | *-* |
| *Dh44^Mi^* | 6 | 0.13 ±0.02 | *P*=4.8X10^-10^ | *-* |
| *trpγ^1^* | 12 | -0.12 ±0.09 | *P*=2.9X10^-5^ | *-* |
| *trpγ ^G4^* | 6 | -0.11 ±0.10 | *P*=9.9X10^-4^ | *-* |
| *trpγ ^1^*;*g*(*trpγ*) | 6 | -0.56 ±0.04 | *P*=0.69 | *P*=3.3X10^-4^ |
| *trpγ^1^*,*UAS*-*trpγ*/*trpγ^1^* | 7 | -0.10 ±0.05 | *P*=6.8X10^-6^ | *P*=0.77 |
| *trpγ^1^*,*UAS*-*trpγ*/*trpγ^G4^* | 6 | -0.52 ±0.11 | *P*=0.34 | *P*=1.5X10^-4^ |

**Figure 1C** Summary statistics.

| Genotypes | n | Means ±SEMs | *P* values |
| --- | --- | --- | --- |
| control | 6 | -0.57 ±0.04 | - |
| *trpγ^1^* | 6 | -0.07 ±0.21 | *P*=4.3X10^-5^ |
| *trpl^29134^* | 6 | -0.47 ±0.12 | *P*=0.15 |
| *iav^3621^* | 6 | -0.46 ±0.30 | *P*=0.08 |
| *nan^36a^* | 6 | -0.47 ±0.02 | *P*=0.07 |
| *trpA1^1^* | 6 | -0.62 ±0.05 | *P*=0.96 |
| *pain^2^* | 6 | -0.55 ±0.03 | *P*=0.42 |
| *pyx^3^* | 6 | -0.48 ±0.09 | *P*=0.16 |
| *amo^1^* | 6 | -0.52 ±0.10 | *P*=0.29 |
| *trpml^2^* | 6 | -0.48 ±0.02 | *P*=0.07 |
| *wtrw^ex^* | 6 | -0.62 ±0.08 | *P*=0.93 |
| *trp^343^* | 6 | -0.67±0.007 | *P*=0.84 |

**Figure 1H** Summary statistics.

| Genotypes | Temperature (17°C →17°C) | | | Temperature (17°→ 31°C) | | |
| --- | --- | --- | --- | --- | --- | --- |
|  | n | Means ±SEMs | *P* values | n | Means ±SEMs | *P* values |
| control | 5 | -0.51 ±0.04 | - | 6 | -0.56 ±0.06 | *P*=0.49 |
| *UAS*-*Kir2*.*1*/+ | 6 | -0.55 ±0.02 | *P*=0.46 | 6 | 0.50 ±0.08 | *P*=0.48 |
| *trpγ^G4^*,*tub*-*GAL80^ts^* | 5 | -0.53 ±0.05 | *P*=0.97 | 5 | -0.49 ±0.10 | *P*=0.94 |
| *trpγ^G4^*,*tub*-*GAL80^ts^*,*UAS*-*Kir2*.*1* | 6 | -0.48 ±0.06 | *P*=0.66 | 5 | -0.062 ±0.08 | *P*=1.4 X10^-3^ |

**Figure 1I** Summary statistics.

| Genotypes | n | Means ±SEMs | *P* values  (with control) | *P* values  (with *trpγ^1^*) |
| --- | --- | --- | --- | --- |
| control | 10 | -0.51 ±0.03 | - | - |
| *trpγ^1^* | 12 | 0.13 ±0.02 | *P*=2.9X10^-5^ | *-* |
| *trpγ^1^*,*UAS*-*trpγ*/*trpγ^1^* | 7 | -0.10 ±0.05 | *P*=6.8X10^-6^ | *P*=0.77 |
| *trpγ^1^*;*Dh44*-*GAL4*/+ | 6 | -0.11 ±0.03 | *P*=7.8X10^-8^ | *P*=0.77 |
| *trpγ^1^*;*iav*-*GAL4*/+ | 6 | -0.14 ±0.01 | *P*=1.6 X10^-4^ | *P*=0.97 |
| *trpγ^1^*,*dilp2*-*GAL4* | 6 | -0.11 ±0.05 | *P*=1.9X10^-3^ | *P*=0.99 |
| *trpγ^1^*,*UAS*-*trpγ*/*trpγ^1^*;*Dh44*-*GAL4*/+ | 6 | -0.51 ±0.01 | *P*=0.99 | *P*=9.9X10^-3^ |
| *trpγ^1^*,*UAS-trpγ*/*trpγ^1^*;*iav*-*GAL4*/+ | 6 | -0.19 ±0.05 | *P*=0.87 | *P*=0.94 |
| *trpγ^1^*,*UAS-trpγ*/*trpγ^1^*,*dilp2*-*GAL4* | 6 | -0.24 ±0.02 | *P*=0.19 | *P*=0.60 |

**Figure 2C** Summary statistics.

| Genotypes | n  (D-glu, L-glu) | Means ±SEMs D-glucose | *P* value  (with control) | Means ±SEMs  (L-glucose) | *P* value  (D-glu vs L-glu) |
| --- | --- | --- | --- | --- | --- |
| control | (16, 10) | 2.5 ±0.20 | *-* | 0.5 ±0.15 | *P*=3.1X10^-7^ |
| *trpγ^1^*/+ | (11, 11) | 2.8 ±0.31 | *P*=0.42 | *-* | *-* |
| *trpγ^1^* | (20, 10) | 4.7 ±0.36 | *P*=1.8 X10^-6^ | 0.4 ±0.07 | *P*=6.1X10^-10^ |

*Dh44>GCaMP6s*

**Figure 2F** Summary statistics.

| Genotypes | n  (D-glu, L-glu) | Means ±SEMs D-glucose | *P* value  (with control) | Means ±SEMs  (L-glucose) | *P* value  (D-glu vs L-glu) |
| --- | --- | --- | --- | --- | --- |
| control | (16, 10) | 0.025 ±0.01 | *-* | 0.033 ±0.01 | *P*=0.06 |
| *trpγ^1^*/+ | (11, 11) | 0.033 ±0.01 | *P*=0.09 | *-* | *-* |
| *trpγ^1^* | (20, 9) | 0.035 ±0.01 | *P*=0.27 | 0.026 ±0.01 | *P*=0.26 |

*Dh44>GCaMP6s*

**Figure 3A** Summary statistics.

| Genotypes/conditions | n | Means ±SEMs D-glucose | *P* value  (vs control with Ca^2+^) |
| --- | --- | --- | --- |
| control (with Ca^2+^) | 5 | 14.6 ±1.41 | - |
| control (Ca^2+^ free) | 5 | 24.3 ±2.19 | *P*=3.6X10^-3^ |
| *trpγ^1^* (with Ca^2+^) | 5 | 22.5 ±3.99 | *P*=0.04 |
| *trpγ^1^* (Ca^2+^ free) | 5 | 31.5 ±3.61 | *P*=2.0X10^-3^ |

*Dh44>ASAP2*

**Figure 3B** Summary statistics.

| Genotypes | n | Means ±SEMs D-glucose | *P* value  (with control buffer) | *P* value  (with control D-glu) |
| --- | --- | --- | --- | --- |
| control (buffer with Ca^2+^) | 5 | 4.1 ±0.65 | *-* | - |
| control (D-glu with Ca^2+^) | 5 | 9.5 ±0.68 | *P*=6.3X10^-4^ | - |
| control (L-glu with Ca^2+^) | 7 | 4.7 ±0.66 | *P*=0.51 | - |
| control (D-glu Ca^2+^ free buffer) | 5 | 13.6 ±1.17 | - | *P*=0.02 |
| *trpγ^1^* (buffer with Ca^2+^) | 5 | 4.1 ±0.76 | - | - |
| *trpγ^1^* (D-glu with Ca^2+^) | 5 | 13.4 ±1.40 | - | *P*=0.04 |
| *trpγ^1^* (L-glu with Ca^2+^) | 5 | 5.6 ±0.54 | *P*=0.11 | - |
| *trpγ^1^* (D-glu Ca^2+^ free buffer) | 5 | 13.9 ±1.80 | - | *P*=0.04 |

*Dh44>ASAP2*

**Figure 3C** Summary statistics.

| Genotypes/conditions | n | Means ±SEMs D-glucose | *P* value  (vs control with Ca^2+^) |
| --- | --- | --- | --- |
| control (with Ca^2+^) | 5 | 7.6 ±1.81 | - |
| control (Ca^2+^ free) | 5 | 5.3 ±1.39 | *P*=0.34 |
| *trpγ^1^* (with Ca^2+^) | 5 | 3.8 ±0.53 | *P*=0.08 |
| *trpγ^1^* (Ca^2+^ free) | 5 | 5.9 ±0.96 | *P*=0.43 |

*Dh44>ASAP2*

**Figure 4A** Summary statistics.

| Genotypes | sated | | | starved | | |
| --- | --- | --- | --- | --- | --- | --- |
|  | n | Means ±SEMs | *P* values | n | Means  ±SEMs | *P* values |
| control | 10 | 2.1 ±0.02 | - | 8 | 1.2 ±0.03 | - |
| *trpγ^1^* | 10 | 2.1 ±0.03 | *P*=0.57 | 7 | 0.9 ±0.07 | *P*=0.09 |
| *trpγ ^G4^* | 8 | 1.1 ±0.04 | *P*=0.09 | 8 | 0.9 ±0.03 | *P*=0.08 |
| *Dh44^Mi^* | 8 | 2.1 ±0.04 | *P*=0.98 | 7 | 1.0 ±0.04 | *P*=0.09 |
| *trpγ^1^*,*UAS*-*trpγ*/*trpγ^1^* | 8 | 2.1 ±0.07 | *P*=0.92 | 8 | 1.0 ±0.03 | *P*=0.13 |
| *trpγ^1^*,*UAS*-*trpγ*/*trpγ^G4^* | 8 | 2.1 ±0.06 | *P*=0.31 | 8 | 1.1 ±0.02 | *P*=0.23 |

**Figure 4B** Summary statistics.

| Genotypes | sated | | | |
| --- | --- | --- | --- | --- |
|  | n | Means ±SEMs | *P* values  (with control) | *P* values  (with *trpγ^1^*) |
| control | 6 | 8.4 ±0.25 | - | - |
| *Dh44^Mi^* | 6 | 4.1 ±0.12 | *P*=9.7X10^-6^ | - |
| *trpγ^1^* | 6 | 4.2 ±0.08 | *P*=2.5X10^-7^ | - |
| *trpγ ^G4^* | 6 | 5.2 ±0.37 | *P*=3.9X10^-4^ | *P*=0.21 |
| *trpγ^1^*,*UAS-trpγ*/*trpγ^1^* | 6 | 3.8 ±0.22 | *P*=2.4X10^-6^ | *P*=0.57 |
| *trpγ^1^*;*Dh44*-*GAL4*/+ | 6 | 4.5 ±0.35 | *P*=5.0X10^-5^ | *P*=0.46 |
| *trpγ^1^*,*dilp2*-*GAL4* | 6 | 5.1 ±0.32 | *P*=1.2X10^-3^ | *P*=0.14 |
| *trpγ^1^*,*UAS*-*trpγ*/*trpγ^G4^* | 6 | 7.8 ±0.44 | *P*=0.32 | *P*=1.3X10^-5^ |
| *trpγ^1^*,*UAS-trpγ*/  *trpγ^1^*;*Dh44*-*GAL4*/+ | 6 | 7.2 ±0.40 | *P*=0.06 | *P*=4.2X10^-5^ |
| *trpγ^1^*,*UAS*-*trpγ*/*trpγ^1^*  ,*dilp2*-*GAL4* | 6 | 5.9 ±0.39 | *P*=3.7X10^-4^ | *P*=0.30 |
| *trpγ^1^*;*g*(*trpγ^1^*) | 6 | 7.8 ±0.34 | *P*=0.21 | *P*=1.8 X10^-6^ |

| Genotypes | starved | | | |
| --- | --- | --- | --- | --- |
|  | n | Means  ±SEMs | *P* values  (with control) | *P* values  (with *trpγ^1^*) |
| control | 6 | 3.7 ±0.31 | - | - |
| *Dh44^Mi^* | 6 | 1.0 ±0.07 | *P*=1.1X10^-4^ | *-* |
| *trpγ^1^* | 6 | 1.8 ±0.16 | *P*=9.2X10^-4^ | *-* |
| *trpγ ^G4^* | 6 | 1.8 ±0.26 | *P*=0.013 | *P*=0.88 |
| *trpγ^1^*,*UAS*-*trpγ*/*trpγ^1^* | 7 | 1.2 ±0.09 | *P*=4.8X10^-5^ | *P*=0.12 |
| *trpγ^1^*;*Dh44*-*GAL4*/+ | 7 | 1.7 ±0.10 | *P*=3.5X10^-4^ | *P*=0.87 |
| *trpγ^1^*,*dilp2*-*GAL4* | 6 | 2.1 ±0.13 | *P*=0.05 | *P*=0.23 |
| *trpγ^1^*,*UAS*-*trpγ*/*trpγ^G4^* | 7 | 3.6 ±0.16 | *P*=0.30 | *P*=2.2X10^-3^ |
| *trpγ^1^*,*UAS*-*trpγ*/  *trpγ^1^*;*Dh44*-*GAL4*/+ | 6 | 3.7 ±0.29 | *P*=0.37 | *P*=4.4X10^-3^ |
| *trpγ^1^*,*UAS*-*trpγ*/*trpγ^1^*  ,*dilp2*-*GAL4* | 6 | 2.7 ±0.24 | *P*=0.02 | *P*=0.21 |
| *trpγ^1^*;*g*(*trpγ^1^*) | 7 | 3.7 ±0.38 | *P*=0.38 | *P*=3.4X10^-4^ |

**Figure 4C** Summary statistics.

| Genotypes | sated | | | |
| --- | --- | --- | --- | --- |
|  | n | Means ±SEMs | *P* values  (with control) | *P* values  (with *trpγ^1^*) |
| control | 6 | 13.4 ±0.71 | - | - |
| *Dh44^Mi^* | 6 | 8.8 ±0.85 | *P*=9.8X10^-3^ | - |
| *trpγ^1^* | 6 | 9.7 ±0.96 | *P*=6.4X10^-3^ | - |
| *trpγ ^G4^* | 6 | 9.1 ±0.61 | *P*=0.01 | *P*=0.78 |
| *trpγ^1^*,*UAS*-*trpγ*/*trpγ^1^* | 6 | 9.0 ±1.27 | *P*=9.7X10^-3^ | *P*=0.35 |
| *trpγ^1^*;*Dh44*-*GAL4*/+ | 6 | 9.7 ±0.62 | *P*=4.4X10^-3^ | *P*=0.44 |
| *trpγ^1^*,*dilp2*-*GAL4* | 6 | 10.8 ±0.49 | *P*=9.2 X10^-3^ | *P*=0.14 |
| *trpγ^1^*,*UAS*-*trpγ*/*trpγ^G4^* | 6 | 13.8 ±0.72 | *P*=0.89 | *P*=0.01 |
| *trpγ^1^*,*UAS*-*trpγ*/  *trpγ^1^*;*Dh44*-*GAL4*/+ | 6 | 13.8 ±0.92 | *P*=0.91 | *P*=6.3X10^-3^ |
| *trpγ^1^*,*UAS*-*trpγ*/*trpγ^1^*  ,*dilp2*-*GAL4* | 6 | 10.9 ±0.49 | *P*=0.21 | *P*=0.56 |
| *trpγ^1^*;*g*(*trpγ^1^*) | 6 | 13.1 ±0.53 | *P*=0.96 | *P*=2.4 X10^-3^ |

| Genotypes | starved | | | |
| --- | --- | --- | --- | --- |
|  | n | Means ±SEMs | *P* values  (with control) | *P* values  (with *trpγ^1^*) |
| control | 7 | 6.6 ±0.54 | - | - |
| *Dh44^Mi^* | 8 | 3.4 ±0.53 | *P* =4.9X10^-4^ | *-* |
| *trpγ^1^* | 6 | 3.2 ±0.08 | *P* =7.1X10^-4^ | *-* |
| *trpγ ^G4^* | 6 | 3.3 ±0.15 | *P*=2.6X10^-3^ | *P*=0.73 |
| *trpγ^1^*,*UAS*-*trpγ*/*trpγ^1^* | 6 | 3.4 ±0.53 | *P*=8.1X10^-3^ | *P*=0.90 |
| *trpγ^1^*;*Dh44*-*GAL4*/+ | 6 | 3.3 ±0.30 | *P*=1.9X10^-3^ | *P*=0.87 |
| *trpγ^1^*,*dilp2*-*GAL4* | 6 | 3.4 ±0.34 | *P*=0.47 | *P*=0.75 |
| *trpγ^1^*,*UAS*-*trpγ*/*trpγ^G4^* | 8 | 7.5 ±0.44 | *P*=0.84 | *P*=2.6 X10^-4^ |
| *trpγ^1^*,*UAS*-*trpγ*/  *trpγ^1^*;*Dh44*-*GAL4*/+ | 6 | 6.2 ±0.34 | *P*=0.46 | *P*=2.4 X10^-4^ |
| *trpγ^1^*,*UAS*-*trpγ*/*trpγ^1^*  ,*dilp2*-*GAL4* | 6 | 4.6 ±0.36 | *P*=0.01 | *P*=0.35 |
| *trpγ^1^*;*g*(*trpγ^1^*) | 6 | 7.0 ±0.42 | *P*=0.98 | *P*=2.7 X10^-4^ |

**Figure 5C** Summary statistics.

| Genotypes | n | Time (min) | Means ±SEMs | *P* values |
| --- | --- | --- | --- | --- |
| control | 8 | 0 | 0.33 ±0.01 | - |
|  | 11 | 5 | 0.16 ±0.02 | - |
|  | 10 | 15 | 0.18 ±0.01 | - |
|  | 11 | 30 | 0.18 ±0.01 | - |
|  | 11 | 60 | 0.18 ±0.01 | - |
|  | 11 | 90 | 0.16 ±0.01 | - |
|  | 11 | 120 | 0.16 ±0.01 | - |
|  | 11 | 150 | 0.16 ±0.01 | - |
| *trpγ^1^* | 8 | 0 | 0.04 ±0.01 | *P*=0.79 |
|  | 10 | 5 | 0.20 ±0.01 | *P*=5.0X10^-3^ |
|  | 10 | 15 | 0.27 ±0.01 | *P*=7.6X10^-5^ |
|  | 10 | 30 | 0.27 ±0.01 | *P*=2.1X10^-5^ |
|  | 10 | 60 | 0.24 ±0.01 | *P*=1.4X10^-4^ |
|  | 11 | 90 | 0.21 ±0.01 | *P*=9.2X10^-3^ |
|  | 10 | 120 | 0.19 ±0.02 | *P*=0.08 |
|  | 10 | 150 | 0.19 ±0.01 | *P*=0.06 |
| *Dh44* | 8 | 0 | 0.03 ±0.01 | *P*=0.98 |
|  | 9 | 5 | 0.15 ±0.01 | *P*=0.97 |
|  | 11 | 15 | 0.16 ±0.01 | *P*=0.89 |
|  | 10 | 30 | 0.19 ±0.01 | *P*=0.70 |
|  | 10 | 60 | 0.17 ±0.01 | *P*=0.98 |
|  | 10 | 90 | 0.16 ±0.01 | *P*=0.98 |
|  | 10 | 120 | 0.17 ±0.01 | *P*=0.98 |
|  | 10 | 150 | 0.17 ±0.01 | *P*=0.97 |

**Figure 5D** Summary statistics.

| Genotypes | sated | | | starved | | |
| --- | --- | --- | --- | --- | --- | --- |
|  | n | Means ±SEMs | *P* values | n | Means ±SEMs | *P* values |
| control | 10 | 0.16 ±0.01 | - | 10 | 0.18 ±0.01 | - |
| *trpγ^1^* | 10 | 0.25 ±0.02 | *P*=3.2X10^-4^ | 10 | 0.26 ±0.01 | *P*=5.7X10^-5^ |
| *Dh44^Mi^* | 10 | 0.18 ±0.01 | *P*=0.27 | 10 | 0.19 ±0.01 | *P*=0.73 |

**Figure 5E** Summary statistics.

| Temperature (17°C→17°C) | | | | Temperature (17°C→31°C) | | |
| --- | --- | --- | --- | --- | --- | --- |
| Genotypes | n | Means ±SEMs | *P* values | n | Means ±SEMs | *P* values |
| control | 10 | 0.17 ±0.01 | - | 10 | 0.18 ±0.02 | *P*=0.55 |
| *UAS*-*Kir2*.*1*/+ | 10 | 0.19 ±0.01 | *P*=0.16 | 10 | 0.19 ±0.01 | *P*=0.89 |
| *trpγ^G4^*,*tub*-*GAL80^ts^* | 10 | 0.20 ±0.02 | *P*=0.07 | 10 | 0.20 ±0.03 | *P*=0.72 |
| *trpγ^G4^*,*tub*-*GAL80^ts^*,*UAS*-*Kir2*.*1* | 10 | 0.20 ±0.01 | *P*=0.07 | 10 | 0.26 ±0.02 | *P*=3.1X10^-3^ |

**Figure 5F** Summary statistics.

| Genotypes | n | Means  ±SEMs | *P* values  (with control) | *P* values  (with *trpγ^G4^*) |
| --- | --- | --- | --- | --- |
| control | 15 | 0.16 ±0.01 | - | - |
| *trpγ^1^*,*UAS*-*trpγ*/*trpγ^1^* | 9 | 0.21 ±0.02 | *P*=5.5X10^-4^ | *P*=0.07 |
| *trpγ^G4^* | 10 | 0.22 ±0.01 | *P*=2.4X10^-5^ | *-* |
| *trpγ^1^*;*Dh44*-*GAL4*/+ | 8 | 0.23 ±0.02 | *P*=1.6X10^-5^ | *P*=0.90 |
| *trpγ^1^*,*UAS-trpγ*/*trpγ^G4^* | 14 | 0.17 ±0.01 | *P*=0.46 | *P*=6.7X10^-7^ |
| *trpγ^1^*,*UAS*-*trpγ*/*trpγ^1^*;*Dh44GAL4*/+ | 14 | 0.18 ±0.01 | *P*=0.09 | *P*=8.5X10^-8^ |
| *trpγ^1^*;*g*(*trpγ*) | 10 | 0.17 ±0.02 | *P*=0.07 | *P*=7.7 X10^-4^ |

**Figure 5G** Summary statistics.

| Genotypes | n | Means ±SEMs | *P* values |
| --- | --- | --- | --- |
| control | 10 | 0.17 ±0.01 | - |
| *UAS*-*Kir2.1*/+ | 10 | 0.17 ±0.01 | *P*=0.98 |
| *Dh44*-*GAL4*/+ | 10 | 0.17 ±0.09 | *P*=0.66 |
| *UAS*-*Kir2.1*/+;*Dh44*-*GAL4*/+ | 10 | 0.27 ±0.01 | *P*=2.4X10^-6^ |

**Figure 6B** Summary statistics.

| Genotypes | n | Crop score | Means (%) ±SEMs | *P* values  (with control) | *P* values  (with *trpγ^1^*) |
| --- | --- | --- | --- | --- | --- |
| control | 5 | 1 | 9.2 ±1.78 | - | - |
|  | 5 | 2 | 13.2 ±4.09 | - | - |
|  | 5 | 3 | 55.8 ±2.10 | - | - |
|  | 5 | 4 | 12.7 ±4.58 | - | - |
|  | 5 | 5 | 9.7 ±2.74 | - | - |
| *trpγ^1^* | 5 | 1 | 10.2 ±2.59 | *P*=0.79 | *-* |
|  | 5 | 2 | 7.5 ±1.57 | *P*=0.56 | *-* |
|  | 5 | 3 | 34.7 ±1.96 | *P*=2.5X10^-3^ | *-* |
|  | 5 | 4 | 18.8 ±1.30 | *P*=0.96 | *-* |
|  | 5 | 5 | 28.9 ±2.74 | *P*=1.9 X10^-3^ | *-* |
| *trpγ^1^*,*UAS*-*trpγ*/*trpγ^G4^* | 5 | 1 | 5.7 ±2.54 | *P*=0.81 | *P*=0.77 |
|  | 5 | 2 | 11.7 ±2.13 | *P*=0.99 | *P*=0.99 |
|  | 5 | 3 | 53.9 ±3.55 | *P*=0.99 | *P*=1.2X10^-4^ |
|  | 5 | 4 | 14.9 ±1.16 | *P*=0.78 | *P*=0.90 |
|  | 5 | 5 | 17.0 ±2.14 | *P*=0.21 | *P*=0.66 |
| *trpγ^1^*,*UAS*-*trpγ*/  *trpγ^1^*;*Dh44*-*GAL4*/+ | 5 | 1 | 9.0 ±2.78 | *P*=0.99 | *P*=9.95 |
|  | 5 | 2 | 9.4 ±0.32 | *P*=0.98 | *P*=0.99 |
|  | 5 | 3 | 52.4 ±4.62 | *P*=0.81 | *P*=4.7X10^-3^ |
|  | 5 | 4 | 13.3 ±2.14 | *P*=0.99 | *P*=0.97 |
|  | 5 | 5 | 16.1 ±3.02 | *P*=0.52 | *P*=0.57 |
| *trpγ^1^*,*UAS*-*trpγ*/  *trpγ^1^*,*dilp2*-*GAL4* | 5 | 1 | 17.2 ±1.57 | *P*=0.23 | *P*=0.10 |
|  | 5 | 2 | 15.6 ±2.53 | *P*=0.95 | *P*=0.68 |
|  | 5 | 3 | 31.5 ±2.78 | *P*=0.11 | *P*=0.88 |
|  | 5 | 4 | 9.9 ±2.18 | *P*=0.14 | *P*=0.99 |
|  | 5 | 5 | 25.7 ±2.80 | *P*=0.07 | *P*=0.80 |

**Figure 6C** Summary statistics.

| Genotypes | n | Crop score | Means (%) ±SEMs | *P* values |
| --- | --- | --- | --- | --- |
| control D-glu | 5 | 1 | 8.4 ±1.09 | - |
|  | 5 | 2 | 12.5 ±1.11 | - |
|  | 5 | 3 | 55.4 ±2.84 | - |
|  | 5 | 4 | 10.7 ±3.01 | - |
|  | 5 | 5 | 13.0 ±1.24 | - |
| control L-glu | 5 | 1 | 10.6 ±2.29 | *P*=0.73 |
|  | 5 | 2 | 9.8 ±2.14 | *P*=0.89 |
|  | 5 | 3 | 38.9 ±2.16 | *P*=6.6X10^-3^ |
|  | 5 | 4 | 13.4 ±1.30 | *P*=0.50 |
|  | 5 | 5 | 27.4 ±1.93 | *P*=3.1X10^-3^ |
| *trpγ^1^* D-glu | 5 | 1 | 9.3 ±0.83 | - |
|  | 5 | 2 | 8.5 ±2.16 | - |
|  | 5 | 3 | 33.2 ±2.34 | - |
|  | 5 | 4 | 16.8 ±1.90 | - |
|  | 5 | 5 | 32.8 ±2.82 | - |
| *trpγ^1^* L-glu | 5 | 1 | 8.3 ±3.27 | *P*=0.59 |
|  | 5 | 2 | 10.2 ±2.47 | *P*=0.97 |
|  | 5 | 3 | 25.8 ±2.35 | *P*=0.30 |
|  | 5 | 4 | 14.6 ±2.90 | *P*=1.1X10^-4^ |
|  | 5 | 5 | 41.3 ±5.73 | *P*=0.60 |

**Figure 6D** Summary statistics.

| Genotypes | n | Means ±SEMs | *P* values  (with control) | *P* values  (with *trpγ^1^*) |
| --- | --- | --- | --- | --- |
| control | 39 | 20.2 ±0.47 | - | - |
| *trpγ^1^* | 26 | 25.7 ±0.85 | *P*=6.4X10^-4^ | *-* |
| *Dh44^Mi^* | 22 | 18.4 ±0.71 | *P*=0.59 | *-* |
| *trpγ ^G4^* | 14 | 25.3 ±1.22 | *P*=2.0X10^-4^ | *P*=0.80 |
| *trpγ^1^*,*UAS*-*trpγ*/*trpγ^1^* | 23 | 23.9 ±0.70 | *P*=3.6X10^-4^ | *P*=0.10 |
| *trpγ^1^*,*UAS*-*trpγ*/*trpγ^G4^* | 20 | 19.0 ±0.47 | *P*=0.13 | *P*=9.2X10^-5^ |
| *trpγ^1^*;*g*(*trpγ*) | 24 | 21.0 ±0.79 | *P*=0.68 | *P*=1.4X10^-4^ |
| *trpγ^1^*,*UAS*-*trpγ*/  *trpγ^1^*,*dilp2*-*GAL4* | 15 | 22.0 ±1.10 | *P*=0.56 | *P*=0.07 |
| *trpγ^1^*,*UAS*-*trpγ*/*trpγ^1^*;*Dh44*-*GAL4*/+ | 15 | 24.4 ±0.73 | *P*=2.4X10^-3^ | *P*=0.54 |

**Figure 6E** Summary statistics.

| Genotypes | control | | *trpγ^1^* | | *P* values  (control vs *trpγ^1^*) |
| --- | --- | --- | --- | --- | --- |
|  | n | Means  ±SEMs | n | Means  ±SEMs |  |
| Sucrose | 30 | 20.2 ±0.50 | 20 | 25.8 ±0.84 | *P*=6.4X10^-4^ |
| D-glucose | 23 | 19.7 ±0.90 | 15 | 24.4 ±1.13 | *P*=3.7X10^-3^ |
| D-fructose | 16 | 20.6 ±0.95 | 16 | 25.6 ±1.28 | *P*=3.5X10^-3^ |
| D-trehalose | 17 | 18.8 ±1.04 | 17 | 23.8 ±1.16 | *P*=2.0X10^-3^ |
| D-maltose | 16 | 16.7 ±0.60 | 17 | 25.2 ±0.75 | *P*=9.0X10^-7^ |

**Figure 6F** Summary statistics.

| Genotypes | control | | *trpγ^1^* | | *P* values  (control vs *trpγ^1^*) |
| --- | --- | --- | --- | --- | --- |
|  | n | Means ±SEMs | n | Means  ±SEMs |  |
| Crop score 1 | 19 | 15.9 ±0.63 | 18 | 23.8 ±0.90 | *P*=1.5X10^-6^ |
| Crop score 2 | 16 | 16.0 ±0.50 | 18 | 21.2 ±0.55 | *P*=1.9X10^-6^ |
| Crop score 3 | 25 | 22.2 ±1.03 | 17 | 22.5 ±1.25 | *P*=0.92 |
| Crop score 4 | 16 | 16.1 ±0.55 | 18 | 22.2 ±0.83 | *P*=7.3X10^-4^ |
| Crop score 5 | 17 | 12.1 ±0.48 | 19 | 25.0 ±1.46 | *P*=9.1X10^-7^ |

**Figure 6G** Summary statistics.

| Genotypes | D-glucose | | L-glucose | | *P* values  (D-glu vs L-glu) | *P* values (with D-glu control) | *P* values (with L-glu control) |
| --- | --- | --- | --- | --- | --- | --- | --- |
|  | n | Means ±SEMs | n | Means ±SEMs |  |  |  |
| control | 23 | 19.7 ±0.86 | 20 | 15.7 ±0.91 | *P*=6.0X10^-3^ | *-* | *-* |
| *trpγ^1^* | 16 | 24.4 ±1.13 | 17 | 24.7 ±0.57 | *P*=0.92 | *P*=1.0X10^-3^ | *P*=1.6X10^-7^ |
| *Dh44^Mi^* | 20 | 19.6 ±0.59 | 21 | 17.2 ±1.12 | *P*=0.21 | *P*=0.88 | *P*=0.83 |

**Figure 7A** Summary statistics.

| Genotypes | n | Time (min) | Means ±SEMs | *P* values |
| --- | --- | --- | --- | --- |
| control | 9 | 15 | 0.7 ±0.75 | - |
|  | 9 | 20 | 6.5 ±1.50 | - |
|  | 9 | 25 | 14.7 ±2.92 | - |
|  | 9 | 30 | 26.2 ±4.40 | - |
|  | 9 | 35 | 35.7 ±5.93 | - |
|  | 9 | 40 | 38.2 ±5.50 | - |
|  | 9 | 45 | 41.7 ±5.54 | - |
|  | 9 | 55 | 44.5 ±6.33 | - |
|  | 9 | 65 | 45.5 ±6.71 | - |
|  | 9 | 75 | 45.5 ±6.71 | - |
| *trpγ^1^* | 9 | 15 | 3.00 ±1.04 | *P*=0.91 |
|  | 9 | 20 | 9.10 ±4.25 | *P*=0.88 |
|  | 9 | 25 | 21.1 ±9.22 | *P*=0.67 |
|  | 9 | 30 | 36.3 ±11.3 | *P*=9.2X10^-3^ |
|  | 9 | 35 | 50.0 ±9.22 | *P*=6.2X10^-3^ |
|  | 9 | 40 | 55.0 ±8.10 | *P*=9.0X10^-3^ |
|  | 9 | 45 | 58.0 ±7.50 | *P*=7.2X10^-3^ |
|  | 9 | 55 | 60.1 ±7.05 | *P*=5.2X10^-3^ |
|  | 9 | 65 | 61.0 ±7.02 | *P*=2.0X10^-3^ |
|  | 9 | 75 | 61.0 ±7.05 | *P*=3.4X10^-3^ |
| *Dh44^Mi^* | 9 | 15 | 0.50 ±0.50 | *P*=0.98 |
|  | 9 | 20 | 6.00 ±1.78 | *P*=0.89 |
|  | 9 | 25 | 13.7 ±3.06 | *P*=0.97 |
|  | 9 | 30 | 23.3 ±5.74 | *P*=0.63 |
|  | 9 | 35 | 24.7 ±4.22 | *P*=9.2X10^-3^ |
|  | 9 | 40 | 25.5 ±4.56 | *P*=8.1X10^-3^ |
|  | 9 | 45 | 27.7 ±4.57 | *P*=4.7X10^-3^ |
|  | 9 | 55 | 30.5 ±4.37 | *P*=4.4X10^-3^ |
|  | 9 | 65 | 30.7 ±4.60 | *P*=1.1X10^-4^ |
|  | 9 | 75 | 31.0 ±4.60 | *P*=1.1X10^-3^ |

**Figure 7B** Summary statistics.

| Genotypes | n | Means  ±SEMs | *P* values  (with control) | *P* values  (with *trpγ^1^*) |
| --- | --- | --- | --- | --- |
| control | 10 | 1.9 ±0.31 | - | - |
| *Dh44^Mi^* | 10 | 1.4 ±0.33 | *P*=7.2X10^-3^ | *-* |
| *trpγ^1^* | 12 | 2.7 ±0.27 | *P*=1.4X10^-4^ | *-* |
| *trpγ ^G4^* | 8 | 2.5 ±0.45 | *P*=5.3X10^-3^ | *P*=0.80 |
| *trpγ^1^*,*UAS*-*trpγ*/*trpγ^1^* | 8 | 2.5 ±0.37 | *P*=7.1X10^-3^ | *P*=0.62 |
| *trpγ^1^*;*Dh44*-*GAL4*/+ | 9 | 2.4 ±0.22 | *P*=8.0X10^-3^ | *P*=0.22 |
| *trpγ^1^*,*dilp2*-*GAL4* | 8 | 2.8 ±0.31 | *P*=2.2X10^-3^ | *P*=0.99 |
| *trpγ^1^*,*UAS*-*trpγ*/*trpγ^G4^* | 9 | 2.0 ±0.19 | *P*=0.96 | *P*=2.1X10^-4^ |
| *trpγ^1^*,*UAS*-*trpγ*/  *trpγ^1^*;*Dh44*-*GAL4*/+ | 9 | 2.4 ±0.37 | *P*=0.13 | *P*=0.42 |
| *trpγ^1^*,*UAS-trpγ*/*trpγ^1^*  ,*dilp2*-*GAL4* | 8 | 3.0 ±0.23 | *P*=0.08 | *P*=0.87 |
| *trpγ^1^*;*g*(*trpγ^1^*) | 9 | 1.7 ±0.35 | *P*=0.71 | *P*=4.4X10^-4^ |

**Figure 7C** Summary statistics.

| Genotypes | n | Means  ±SEMs | *P* values  (with control) | *P* values  (with *trpγ^1^*) |
| --- | --- | --- | --- | --- |
| control | 20 | 1.5 ±0.13 | - | - |
| *trpγ^1^* | 20 | 3.1 ±0.21 | *P*=1.6X10^-6^ | *-* |
| *Dh44^Mi^* | 20 | 2.5 ±0.18 | *P*=4.4X10^-6^ | *-* |
| *trpγ ^G4^* | 20 | 2.2 ±0.18 | *P*=1.4X10^-4^ | *P*=0.68 |
| *trpγ^1^*,*UAS*-*trpγ*/*trpγ^1^* | 20 | 2.4 ±0.23 | *P*=2.2X10^-6^ | *P*=0.5 |
| *trpγ^1^*,*UAS*-*trpγ*/*trpγ^G4^* | 20 | 1.5 ±0.18 | *P*=0.80 | *P*=1.2X10^-6^ |
| *trpγ^1^*;*g*(*trpγ*) | 20 | 1.5 ±0.18 | *P*=0.96 | *P*=9.9X10^-8^ |
| *trpγ^1^*,*UAS*-*trpγ*/*trpγ^1^*,*dilp2*-*GAL4* | 20 | 2.3 ±0.14 | *P*=1.4X10^-6^ | *P*=0.17 |
| *trpγ^1^*,*UAS*-*trpγ*/*trpγ^1^*;*Dh44*-*GAL4*/+ | 20 | 2.7 ±0.29 | *P*=5.4X10^-6^ | *P*=0.94 |

**Figure 7D** Summary statistics.

| Genotypes | n | Means ±SEMs | *P* values  (with control) | *P* values  (with *trpγ^1^*) |
| --- | --- | --- | --- | --- |
| control | 8 | 1.3 ±0.07 | - | - |
| *trpγ^1^* | 8 | 2.0 ±0.04 | *P*=7.9X10^-7^ | *-* |
| *Dh44^Mi^* | 8 | 1.5 ±0.02 | *P*=0.22 | *-* |
| *trpγ ^G4^* | 8 | 1.9 ±0.05 | *P*=5.9X10^-5^ | *P*=0.06 |
| *trpγ^1^*,*UAS*-*trpγ*/*trpγ^1^* | 8 | 1.7 ±0.02 | *P*=3.4X10^-4^ | *P*=0.07 |
| *trpγ^1^*,*UAS*-*trpγ*/*trpγ^G4^* | 8 | 1.1 ±0.05 | *P*=0.05 | *P*=9.4X10^-8^ |
| *trpγ^1^*;*g*(*trpγ*) | 8 | 1.4 ±0.03 | *P*=0.58 | *P*=1.2X10^-6^ |
| *trpγ^1^*,*UAS-trpγ*/*trpγ^1^*,*dilp2*-*GAL4* | 8 | 1.9 ±0.02 | *P*=6.7X10^-5^ | *P*=0.72 |
| *trpγ^1^*,*UAS*-*trpγ*/*trpγ^1^*;*Dh44*-*GAL4*/+ | 8 | 1.9 ±0.01 | *P*=1.7X10^-5^ | *P*=0.25 |

**Figure 1-figure supplement 1A** Summary statistics.

| Genotypes | 50 mM D-glucose | | | 200 mM L-glucose | | |
| --- | --- | --- | --- | --- | --- | --- |
|  | n | Means ±SEMs | *P* values | n | Means ±SEMs | *P* values |
| control | 10 | 14.4 ±1.32 | - | 10 | 21.8 ±2.60 | - |
| *trpγ^1^* | 10 | 13.2 ±1.45 | *P*=0.46 | 10 | 22.0 ±1.60 | *P*=0.93 |
| *Gr64^ab^* | 10 | 1.2 ±0.60 | *P*=2.9X10^-9^ | 10 | 1.4 ±0.94 | *P*=1.5X10^-8^ |

**Figure 1-figure supplement 1C** Summary statistics.

| Genotypes | 50 mM D-glucose | | | 200 mM L-glucose | | |
| --- | --- | --- | --- | --- | --- | --- |
|  | n | Means ±SEMs | *P* values | n | Means ±SEMs | *P* values |
| control | 4 | 68.9 ±6.21 | - | 4 | 68.3 ±1.70 | - |
| *trpγ^1^* | 4 | 68.3 ±1.70 | *P*=0.90 | 4 | 73.3 ±4.71 | *P*=0.49 |

**Figure 1-figure supplement 1D** Summary statistics.

| Genotypes | 50 mM D-glucose | | | 200 mM L-glucose | | |
| --- | --- | --- | --- | --- | --- | --- |
|  | n | Means ±SEMs | *P* values | n | Means ±SEMs | *P* values |
| control | 4 | 75.0 ±2.90 | - | 4 | 85.0 ±2.90 | - |
| *trpγ^1^* | 4 | 71.7 ±1.44 | *P*=0.95 | 4 | 79.1 ±4.39 | *P*=0.87 |

**Figure 1-figure supplement 1E** Summary statistics.

| Genotypes | D-glucose | | L-glucose | | *P* values  (D-glu vs L-glu) | *P* values (with D-glu control) | *P* values (with L-glu control) |
| --- | --- | --- | --- | --- | --- | --- | --- |
|  | n | Means ±SEMs | n | Means ±SEMs |  |  |  |
| control | 15 | 1.6 ±0.14 | 15 | 1.9 ±0.22 | *P*=0.33 | - | - |
| *trpγ^1^* | 15 | 1.5 ±0.20 | 15 | 1.4 ±0.18 | *P*=0.73 | *P*=0.70 | *P*=0.12 |

**Figure 1-figure supplement 1F** Summary statistics.

| Genotypes | n | Means ±SEMs | *P* values |
| --- | --- | --- | --- |
| *UAS*-*Kir2.1*/+ | 6 | -0.49 ±0.08 | - |
| *Dh44*-*GAL4*/+ | 6 | -0.53 ±0.11 | *P*=0.98 |
| *UAS*-*Kir2.1*/+;*Dh44-GAL4*/+ | 6 | -0.06 ±0.02 | *P*=8.5X10^-5^ |
| *Dilp2*-*GAL4*/+ | 6 | -0.51 ±0.11 | *P*=0.88 |
| *UAS*-*Kir2.1*,*dilp2*-*GAL4* | 6 | -0.42 ±0.10 | *P*=0.17 |

**Figure 1-figure supplement 1G** Summary statistics.

| Genotypes | n | Means ±SEMs | *P* values |
| --- | --- | --- | --- |
| control | 4 | -0.52 ±0.01 | - |
| *UAS*-*dicer2*/+;*UAS*- *trpγ^RNAi^*/+ | 4 | -0.50 ±0.02 | *P*=0.98 |
| *Dh44*-*GAL4*/+ | 4 | -0.52 ±0.02 | *P*=0.98 |
| *UAS*-*trpγ^RNAi^*/+ | 4 | -0.51 ±0.11 | *P*=0.88 |
| *UAS*-*dicer2*/+;*UAS*- *trpγRNAi*/+;*Dh44*-*GAL4*/+ | 4 | -0.11 ±0.01 | *P*=1.2X10^-4^ |

**Figure 2-figure supplement 1A** Summary statistics.

| D-glucose | n | | Means ±SEMs | | *P* values |
| --- | --- | --- | --- | --- | --- |
|  | control | *trpγ^1^* | control | *trpγ^1^* |  |
| 20 mM | 10 | 10 | 2.3 ±0.52 | 4.3 ±0.27 | *P*=3.8 X10^-3^ |

*Dh44>GCaMP6s*

**Figure 3-figure supplement 1A** Summary statistics.

| Concentrations  (mM) | n | | Means ±SEMs | | *P* values  (control vs *trpγ^1^*) | *P* values  (control 0 vs other controls) |
| --- | --- | --- | --- | --- | --- | --- |
|  | control | *trpγ^1^* | control | *trpγ^1^* |  |  |
| 0 | 23 | 22 | 57.6 ±3.40 | 31.2 ±1.70 | *P*=1.9 X10^-7^ | - |
| D-glu 2.5 | 35 | 32 | 38.2 ±1.80 | 27.3 ±2.16 | *P*=2.2 X10^-4^ | *P*=1.3 X10^-6^ |
| D-glu 20 | 24 | 23 | 42.3 ±2.70 | 28.9 ±1.40 | *P*=4.5 X10^-4^ | *P*=4.6 X10^-3^ |
| D-glu 50 | 32 | 30 | 48.2 ±4.0 | 32.7 ±1.95 | *P*=1.7 X10^-4^ | *P*=0.05 |
| L-glu 2.5 | 20 | 19 | 56.3 ±3.20 | 28.7 ±1.81 | *P*=2.2 X10^-4^ | *P*=0.96 |
| D-fru 2.5 | 21 | 19 | 52.4 ±2.92 | 33.1 ±1.67 | *P*=2.0 X10^-6^ | *P*=0.26 |
| D-fru 20 | 22 | 20 | 46.8 ±2.53 | 33.7 ±2.47 | *P*=6.4 X10^-4^ | *P*=0.04 |
| D-fru 50 | 18 | 19 | 43.8 ±3.65 | 34.3 ±1.32 | *P*=9.0 X10^-3^ | *P*=0.02 |

**Figure 3-figure supplement 1B** Summary statistics.

| Concentrations  (mM) | n | | Means ±SEMs | | *P* values |
| --- | --- | --- | --- | --- | --- |
|  | control | *trpγ^1^* | control | *trpγ^1^* |  |
| 0 | 23 | 22 | 1.0 ±0.06 | 0.54 ±0.03 | *P*=1.9 X10^-7^ |
| D-glu 2.5 | 35 | 32 | 1.0 ±0.05 | 0.54 ±0.06 | *P*=2.2 X10^-4^ |
| D-glu 20 | 24 | 23 | 1.0 ±0.06 | 0.72 ±0.04 | *P*=4.5 X10^-4^ |
| D-glu 50 | 32 | 30 | 1.0 ±0.08 | 0.60 ±0.04 | *P*=1.7 X10^-4^ |
| L-glu 2.5 | 20 | 19 | 1.0 ±0.05 | 0.54 ±0.06 | *P*=2.2 X10^-4^ |
| D-fru 2.5 | 21 | 19 | 1.0 ±0.06 | 0.63 ±0.03 | *P*=2.0 X10^-6^ |
| D-fru 20 | 22 | 20 | 1.0 ±0.05 | 0.72 ±0.05 | *P*=6.4 X10^-4^ |
| D-fru 50 | 18 | 19 | 1.0 ±0.08 | 0.78 ±0.03 | *P*=9.0 X10^-3^ |

**Figure 4-figure supplement 1A** Summary statistics.

| Genotypes | sated | | |
| --- | --- | --- | --- |
|  | n | Means  ±SEMs | *P* values |
| control | 5 | 8.5 ±0.26 | - |
| *UAS*-*dicer2*/+;*UAS-trpγ^RNAi^*/+ | 5 | 8.7 ±0.80 | *P*=0.98 |
| *Dh44*-*GAL4*/+ | 5 | 8.6 ±0.74 | *P*=0.98 |
| *UAS-dicer2*/*+*;*UAS*- *trpγRNAi*/+;*Dh44*-*GAL4*/+ | 5 | 5.7 ±0.56 | *P*=3.6X10^-3^ |

| Genotypes | starved | | |
| --- | --- | --- | --- |
|  | n | Means  ±SEMs | *P* values |
| control | 5 | 4.4 ±0.32 | - |
| *UAS*-*dicer2*/+;*UAS*- *trpγ^RNAi^*/+ | 5 | 4.5 ±0.41 | *P*=0.96 |
| *Dh44*-*GAL4*/+ | 5 | 4.5 ±0.27 | *P*=0.98 |
| *UAS*-*dicer2*/+;*UAS*- *trpγRNAi*/+;*Dh44*-*GAL4*/+ | 5 | 3.0 ±0.30 | *P*=0.018 |

**Figure 4-figure supplement 1B** Summary statistics.

| Genotypes | sated | | |
| --- | --- | --- | --- |
|  | n | Means  ±SEMs | *P* values |
| control | 5 | 13.8 ±1.12 | - |
| *UAS*-*dicer2*/+;*UAS*-*trpγ^RNAi^*/+ | 5 | 13.3 ±0.66 | *P*=0.93 |
| *Dh44-GAL4*/*+* | 5 | 13.6 ±0.81 | *P*=0.99 |
| *UAS*-*dicer2*/+;*UAS*- *trpγRNAi*/+;*Dh44*-*GAL4*/+ | 5 | 9.5 ±0.40 | *P*=0.048 |

| Genotypes | starved | | |
| --- | --- | --- | --- |
|  | n | Means  ±SEMs | *P* values |
| control | 5 | 6.6 ±0.66 | - |
| *UAS*-*dicer2*/+;*UAS*- *trpγ^RNAi^*/+ | 5 | 6.8 ±0.54 | *P*=0.98 |
| *Dh44*-*GAL4*/+ | 5 | 6.1 ±0.41 | *P*=0.90 |
| *UAS*-*dicer2*/+;*UAS*- *trpγRNAi*/+;*Dh44*-*GAL4*/+ | 5 | 3.9 ±0.21 | *P*=0.041 |

**Figure 4-figure supplement 1C** Summary statistics.

| Genotypes | n | LT50 | *P* values  (with control) | *P* values  (with mutant) |
| --- | --- | --- | --- | --- |
| control | 10 | 46.0 ±2.10 | - | - |
| *trpγ^1^* | 10 | 34.0 ±2.90 | *P*=1.4X10^-3^ | *^-^* |
| *trpγ^1^*,*UAS*-*trpγ*/*trpγ^1^* | 10 | 37.7 ±2.84 | *P*=5.2X10^-3^ | *^-^* |
| *trpγ ^G4^* | 10 | 30.0 ±3.21 | *P*=1.5X10^-4^ | *^-^* |
| *trpγ^1^*;*Dh44*-*GAL4/+* | 10 | 36.0 ±3.74 | *P*=2.5X10^-3^ | *^-^* |
| *trpγ^1^*,*UAS*-*trpγ*/*trpγ^G4^* | 10 | 5.0 ±2.28 | *P*=0.86 | *P* =2.5X10^-5^ |
| *trpγ^1^*,*UAS*-*trpγ*/*trpγ^1^*;*Dh44*-*GAL4*/+ | 10 | 49.5 ±3.23 | *P*=0.54 | *P* =3.7X10^-6^ |

**Figure 5-figure supplement 1** Summary statistics.

| Genotypes | D-glucose | | L-glucose | | *P* values  (D-glu vs L-glu) | *P* values (with D-glu control) | *P* values (with L-glu control) |
| --- | --- | --- | --- | --- | --- | --- | --- |
|  | n | Means ±SEMs | n | Means ±SEMs |  |  |  |
| control | 8 | 0.25 ±0.02 | 8 | 0.20 ±0.01 | *P*=2.2X10^-3^ | *-* | *-* |
| *trpγ^1^* | 8 | 0.30 ±0.01 | 8 | 0.30 ±0.03 | *P*=0.72 | *P*=9.1X10^-3^ | *P*=1.4X10^-4^ |
| *Dh44^Mi^* | 8 | 0.23 ±0.03 | 8 | 0.22 ±0.01 | *P*=0.57 | *P*=0.50 | *P*=0.10 |

**Figure 6-figure supplement 1A** Summary statistics.

| Genotypes | n | Crop score | Means (%) ±SEMs | *P* values |
| --- | --- | --- | --- | --- |
| Sucrose | 5 | 1 | 9.2 ±1.02 | - |
|  | 5 | 2 | 13.2 ±2.17 | - |
|  | 5 | 3 | 50.4 ±4.36 | - |
|  | 5 | 4 | 17.0 ±4.87 | - |
|  | 5 | 5 | 9.7 ±2.35 | - |
| D-glucose | 5 | 1 | 9.9 ±1.14 | *P*=0.91 |
|  | 5 | 2 | 12.2 ±1.76 | *P*=0.88 |
|  | 5 | 3 | 55.4 ±4.34 | *P*=0.98 |
|  | 5 | 4 | 8.42 ±3.94 | *P*=0.09 |
|  | 5 | 5 | 13.6 ±2.45 | *P*=0.79 |
| D-fructose | 5 | 1 | 8.42 ±2.60 | *P*=0.99 |
|  | 5 | 2 | 18.3 ±1.91 | *P*=0.89 |
|  | 5 | 3 | 50.9 ±1.52 | *P*=0.97 |
|  | 5 | 4 | 10.0 ±5.12 | *P*=0.08 |
|  | 5 | 5 | 12.1 ±0.60 | *P*=0.88 |

**Figure 6-figure supplement 1B** Summary statistics.

| Genotypes | n | Crop score | Means (%) ±SEMs | *P* values |
| --- | --- | --- | --- | --- |
| control | 5 | 1 | 7.6 ±2.33 | - |
|  | 5 | 2 | 17.7 ±2.61 | - |
|  | 5 | 3 | 54.6 ±3.61 | - |
|  | 5 | 4 | 5.6 ±2.48 | - |
|  | 5 | 5 | 14.6 ±1.33 | - |
| *trpγ^1^* | 5 | 1 | 17.9 ±1.08 | *P*=0.38 |
|  | 5 | 2 | 9.3 ±3.36 | *P*=0.81 |
|  | 5 | 3 | 31.5 ±3.06 | *P*=8.1X10^-3^ |
|  | 5 | 4 | 13.8 ±0.45 | *P*=0.09 |
|  | 5 | 5 | 30.1 ±1.83 | *P*=5.3X10^-3^ |
| *Dh44^Mi^* | 5 | 1 | 16.6 ±1.71 | *P*=0.47 |
|  | 5 | 2 | 16.6 ±1.71 | *P*=0.59 |
|  | 5 | 3 | 35.4 ±2.41 | *P*=1.3X10^-3^ |
|  | 5 | 4 | 12.8 ±0.87 | *P*=0.08 |
|  | 5 | 5 | 18.5 ±2.55 | *P*=0.12 |

**Figure 6-figure supplement 1C** Summary statistics.

| Genotypes | n | Crop score | Means (%) ±SEMs | *P* values |
| --- | --- | --- | --- | --- |
| control | 5 | 1 | 10.5 ±1.55 | - |
|  | 5 | 2 | 14.5 ±2.00 | - |
|  | 5 | 3 | 54.1 ±3.31 | - |
|  | 5 | 4 | 11.5 ±5.87 | - |
|  | 5 | 5 | 9.4 ±3.32 | - |
| *trpγ^1^* | 5 | 1 | 11.9 ±1.90 | *P*=0.37 |
|  | 5 | 2 | 5.7 ±1.25 | *P*=0.64 |
|  | 5 | 3 | 36.9 ±1.37 | *P*=8.3X10^-3^ |
|  | 5 | 4 | 19.8 ±1.39 | *P*=0.21 |
|  | 5 | 5 | 25.7 ±3.41 | *P*=2.2X10^-3^ |
| *Dh44^Mi^* | 5 | 1 | 14.1 ±0.96 | *P*=0.34 |
|  | 5 | 2 | 16.8 ±0.42 | *P*=0.65 |
|  | 5 | 3 | 30.2 ±3.28 | *P*=5.3X10^-3^ |
|  | 5 | 4 | 14.1 ±0.96 | *P*=0.34 |
|  | 5 | 5 | 24.8 ±1.90 | *P*=1.0X10^-3^ |

**Figure 6-figure supplement 1D** Summary statistics.

| Genotypes | n | Crop score | Means (%) ±SEMs | *P* values |
| --- | --- | --- | --- | --- |
| control | 5 | 1 | 9.4 ±1.34 | - |
|  | 5 | 2 | 17.9 ±1.57 | - |
|  | 5 | 3 | 55.7 ±2.99 | - |
|  | 5 | 4 | 6.8 ±1.56 | - |
|  | 5 | 5 | 10.2 ±0.58 | - |
| *trpγ^1^* | 5 | 1 | 19.5 ±0.77 | *P*=0.43 |
|  | 5 | 2 | 11.1 ±1.54 | *P*=0.25 |
|  | 5 | 3 | 32.8 ±2.16 | *P*=1.0X10^-3^ |
|  | 5 | 4 | 13.4 ±0.81 | *P*=0.18 |
|  | 5 | 5 | 22.7 ±1.19 | *P*=4.0X10^-3^ |
| *Dh44^Mi^* | 5 | 1 | 15.3 ±0.87 | *P*=0.68 |
|  | 5 | 2 | 16.2 ±1.73 | *P*=0.65 |
|  | 5 | 3 | 32.0 ±2.16 | *P*=4.3X10^-3^ |
|  | 5 | 4 | 11.3 ±1.83 | *P*=0.22 |
|  | 5 | 5 | 25.2 ±0.83 | *P*=3.7X10^-3^ |

**Figure 6-figure supplement 1E** Summary statistics.

| Genotypes | n | Crop score | Means (%) ±SEMs | *P* values |
| --- | --- | --- | --- | --- |
| control  (sucrose) | 5 | 1 | 9.4 ±1.71 | - |
|  | 5 | 2 | 13.5 ±2.56 | - |
|  | 5 | 3 | 50.4 ±4.36 | - |
|  | 5 | 4 | 16.7 ±4.21 | - |
|  | 5 | 5 | 9.7 ±2.06 | - |
| *Dh44^Mi^* (sucrose) | 5 | 1 | 17.5 ±2.55 | *P*=0.06 |
|  | 5 | 2 | 13.4 ±2.40 | *P*=0.99 |
|  | 5 | 3 | 32.3 ±1.76 | *P*=2.1X10^-3^ |
|  | 5 | 4 | 14.6 ±2.75 | *P*=0.96 |
|  | 5 | 5 | 22.3 ±1.63 | *P*=0.06 |

| Genotypes | n | Crop score | Means (%) ±SEMs | *P* values |
| --- | --- | --- | --- | --- |
| *Dh44^Mi^* D-glu | 5 | 1 | 13.4 ±2.67 | - |
|  | 5 | 2 | 10.3 ±3.94 | - |
|  | 5 | 3 | 32.2 ±2.34 | - |
|  | 5 | 4 | 8.3 ±2.85 | - |
|  | 5 | 5 | 36.8 ±3.12 | - |
| *Dh44^Mi^* L- glu | 5 | 1 | 5.7 ±1.69 | *P*=0.96 |
|  | 5 | 2 | 6.7 ±1.15 | *P*=0.91 |
|  | 5 | 3 | 28.7 ±4.18 | *P*=0.97 |
|  | 5 | 4 | 23.1 ±2.19 | *P*=0.13 |
|  | 5 | 5 | 35.9 ±4.33 | *P*=0.99 |

**Figure 7-figure supplement 1** Summary statistics.

| Genotypes | sated | | starved | | *P* values  (with sated control) | *P* values (with starved control) | *P* values  (sated vs starved) |
| --- | --- | --- | --- | --- | --- | --- | --- |
|  | n | Means ±SEMs | n | Means ±SEMs |  |  |  |
| control | 14 | 5.9 ±0.47 | 14 | 5.2 ±0.30 | - | *-* | *P*=0.34 |
| *trpγ^1^* | 13 | 6.8 ±0.40 | 13 | 5.5 ±0.33 | *P*=0.07 | *P*=0.91 | *P*=0.62 |
| *Dh44^Mi^* | 12 | 6.3 ±0.31 | 12 | 6.8 ±0.28 | *P*=0.65 | *P*=0.22 | *P*=0.46 |
